# Association of pre- and postoperative αKlotho levels with long-term remission after pituitary surgery for acromegaly

**DOI:** 10.1038/s41598-022-19078-8

**Published:** 2022-08-30

**Authors:** Marian Christoph Neidert, Anna Maria Zeitlberger, Henning Leske, Oliver Tschopp, Lisa Sze, Cornelia Zwimpfer, Peter Wiesli, David Bellut, René-Ludwig Bernays, Elisabeth Jane Rushing, Christoph Schmid

**Affiliations:** 1 Department of Neurosurgery, University Hospital Zurich, Zurich, Switzerland; 2 Department of Neurosurgery, Kantonsspital St.Gallen, Rohrschacher Strasse 95, 9007 St. Gallen, Switzerland; 3 Department of Neuropathology, University Hospital Zurich, Zurich, Switzerland; 4 Department of Pathology, Oslo University Hospital, Oslo, Norway; 5 University of Oslo (UiO), Oslo, Norway; 6 Division of Endocrinology and Diabetes, University Hospital Zurich, Zurich, Switzerland; 7 Division of Endocrinology and Diabetes, Kantonsspital Winterthur, Winterthur, Switzerland; 8 Department of Internal Medicine, Kantonsspital Frauenfeld, Frauenfeld, Switzerland; 9 Department of Neurosurgery, Clinic Hirslanden, Zurich, Switzerland

**Keywords:** Endocrine system and metabolic diseases, Neurological disorders

## Abstract

Soluble αKlotho (sKl) is a disease-specific biomarker that is elevated in patients with acromegaly and declines after surgery for pituitary adenoma. Approximately 25% of patients do not achieve remission after surgery, therefore a risk stratification for patients early in the course of their disease may allow for the identification of patients requiring adjuvant treatment. Growth hormone (GH) and insulin-like growth factor-1 (IGF-1) have been assessed as biomarker for disease activity, however the value of sKl as a predictive biomarker of surgical success has not been evaluated yet. In this study, we measured serum biomarkers before and after transsphenoidal pituitary surgery in 55 treatment-naïve patients. Based on biochemical findings at follow-up (7–16 years), we divided patients into three groups: (A) long-term cure (defined by normal IGF-1 and random low GH (< 1 μg/l) or a suppressed GH nadir (< 0.4/μg/l) on oral glucose testing); (B) initial remission with later disease activity; (C) persistent clinical and/or biochemical disease activity. sKl levels positively related to GH, IGF-1 levels and tumor volume. Interestingly, there was a statistically significant difference in pre- and postoperative levels of sKl between the long-term cure group and the group with persistent disease activity. This study provides first evidence that sKl may serve as an additional marker for surgical success, decreasing substantially in all patients with initial clinical remission while remaining high after surgery in patients with persistent disease activity.

## Introduction

Acromegaly is characterized by typical clinical phenotypic and laboratory findings, comprising mainly of elevated growth hormone (GH) levels that are non-suppressible by oral glucose as well as elevated insulin-like growth factor 1 (IGF-1) serum levels. GH excess due to benign pituitary adenomas is the major cause for acromegaly with an annual incidence of approximately 4 cases per 1 million person. Complete removal of the GH-secreting pituitary tumor is the best curative approach, with approximately 75% of patients achieving long-term biochemical remission^1^. Acromegaly is usually diagnosed after considerable delay and possibly therefore associated with increased mortality even after transsphenoidal surgery^2^. Clinical features usually develop slowly and comprise pathognomonic changes in the patient`s appearance such as acral enlargement and coarse facial features due to soft tissue swelling and skeletal bone growth. Metabolic changes include increased plasma glucose in the setting of insulin resistance despite reduced visceral fat, and high levels of serum phosphate accompanied by higher than normal renal glomerular filtration^3,4^. While the underlying mechanisms of these changes are not yet completely understood, both insulin resistance and elevated serum phosphate are known to be associated with increased mortality^5^.

The aging-suppressor gene *α-klotho* was first described in 1997 by Kuro-o et al. Its lifespan-influencing function has been demonstrated in mice models showing that gene disruption results in accelerated ageing^6^, whereas gene overexpression causes lifespan extension^7^. The gene product, α-Klotho protein (αKL), is a type I membrane protein encompassing 1012 amino acids with a large extracellular domain and a short cytoplasmic portion. It is predominantly expressed in the kidneys, choroid plexus and several endocrine organs including the pituitary, parathyroid, testis, ovary, placenta and pancreas^6,8^. The kidney appears to be the principal source of circulating αKL^9^. αKL exists in two forms which are characterized by distinct functions. Membrane-bound αKL is a co-receptor for fibroblast growth factor 23 (FGF23)^10,11^. The extracellular domain of the membrane-bound form can be enzymatically cleaved (ectodomain shedding) and released as soluble αKL into blood, urine and cerebrospinal fluid^12^. Soluble α-Klotho protein (sKl) attenuates insulin as well as IGF-1 signaling and regulates calcium homeostasis^13^. Potential implications of αKL functions, especially in the context of GH and IGF-1 signaling, have been reviewed elsewhere^14^.

To date, GH (a direct product of the adenoma) and IGF-1 (mainly produced in the liver upon GH stimulus) are the classical biochemical markers of disease activity in acromegaly. Furthermore, both GH and IGF-1 have been described as predictive markers for surgical outcome in acromegaly along with tumor volume and cavernous sinus invasion^15^.

Our group previously reported that serum sKl is markedly elevated in patients with acromegaly and that sKl excess is reversed following adenoma removal^14,16^. More recently, a study showed that serum concentrations of sKl correlated with quality of life in patients with acromegaly, supporting the use of sKl as a useful biomarker in acromegaly^17^. However, the use of sKl as a predictive marker for long-term remission has not been assessed yet.

In this study, we investigated changes in sKl before and 1–3 months after surgery as well as the relationship of sKl concentrations with GH and IGF-1 levels. Moreover, we assessed differences in biomarker levels, tumor extension, histological granulation pattern, MIB-1 staining and growth hormone receptor (GHR) phenotypes in patients with different long-term outcomes. The ultimate goal of this study was to assess whether sKl may serve as an additional predictive marker of surgical success in acromegaly.

## Methods

### Patients and study design

We included patients with active, treatment-naïve acromegaly, who underwent primary transsphenoidal surgery from 2003 until 2012 by a single surgeon. The study was approved by the local Institutional Review Board. All subjects gave informed consent. Parts of this patient population have been previously described^16,18–20^. All patients underwent full testing of their pituitary functions, and no patient required replacement with cortisol or thyroxine. The preoperative diagnosis of acromegaly was based on pathognomonic clinical findings and biochemical markers (excess IGF-1 and GH, non-suppressible during a 75 g oral glucose tolerance test (oGTT)). Patients were excluded in case histopathological examination did not identify a GH-producing adenoma, if they had received prior medical treatment for the adenoma or had undergone previous radiotherapy. Tumor volume calculation was based on preoperative magnetic resonance imaging (tumor volume = 4/3π1/2x*1/2y*1/2z). Suprasellar extension was classified using the Hardy classification^21^ and cavernous sinus invasion was assessed according to the Knosp classification^22^. The surgical strategy was transnasal-transsphenoidal using microsurgical technique and intraoperative magnetic resonance imaging (PoleStar N20, 0.15 T, Medtronic Navigation, Minneapolis, MN, USA)^23^.

Patients were follow-up at regular intervals at our outpatient clinic. Based on findings at the last follow-up assessment (7–16 years after surgery), we assigned patients to one of three groups:

- **Group A (cure group):** long-term remission as assessed by clinical follow-up, normal IGF-1 and random low GH (< 1 μg/l) or a suppressed GH nadir (< 0.4/μg/l) on oral glucose testing
- **Group B (recurrence group):** initial biochemical remission (normal range IGF-1), but disease recurrence at later follow-up with no longer suppressible GH
- **Group C (persistence group):** partial resection with persistent disease (clinical and biochemical)

### Assays

GH, IGF-1 and sKl levels were measured at baseline and 1–3 months after surgery in treatment-naïve patients. All blood samples were drawn in the morning after overnight fasting. sKl was determined using a sandwich ELISA, as described by Yamazaki et al*.* (Immuno-Biological Laboratories Co., Ltd Japan)^24^. IGF-1 was measured by radioimmunoassay (RIA), after the removal of carrier proteins, as described elsewhere^16,25^. rhIGF-1 (Chiron/Ciba-Geigy AG, Basel, Switzerland) was used for calibration. GH levels were detected by an immunoradiometric assay (hGH-RIATC; CIS Bio International, Oris Industries, Gif-Sur-Yvette, France). The WHO 2nd international standard 98/574 was used for calibration. GH receptor genotyping was performed as described previously^19,25^.

### Histological work-up

Pituitary adenomas were characterized histologically based on disruption of acinar structure in the Gömöri stain. Immunohistochemical preparations including hGH immunostaining, MIB1 proliferation index and CK8a pattern (sparsely or densely granulated). Since a MIB1 index > 3% is associated with a higher incidence of recurrence in pituitary adenomas, a cut-off of 3% was assigned. GH-producing adenomas were designated as ‘sparsely granulated’ when immunostaining with CK8a highlighted small, round intracytoplasmic fibrous bodies (dot-like staining of intermediate filaments). In contrast, densely granulated adenomas showed a more diffuse, perinuclear or ring-like pattern of immunostaining. Immunostains were performed with the Ventana Optiview detection system (using the following antibodies: MIB1/Ki 67 (clone 30-9, prediluted), growth hormone (polyclonal 1/1000) and CK8a (clone CAM5.2, 1/10).

### Statistics

Statistical analyses were performed using commercially available software IBM SPSS Statistics 22 (SPSS Inc, Chicago, IL, USA) and GraphPad Prism 7 (GraphPad Software Inc, La Jolla, CA, USA). Continuous variables are presented as median with interquartile range (IQR) after confirming the non-Gaussian distribution by Shapiro–Wilk and the Kolmogorov Smirnov test. Biomarker levels before and after surgery were compared using the Wilcoxon signed-rank test and the analysis of variance (ANOVA) as appropriate. Correlation coefficients between sKI and other variables were assessed using Spearman’s rank correlation. The nonparametric Mann–Whitney-U test was performed to compare biomarker levels between male and female patients. Although age difference between groups was not statistically significantly different (*p* = 0.0208), analyses of co-variance (ANCOVA) adjusted for age as a co-variable was used additionally to assess difference between mean biomarker levels between the groups. Using the ANCOVA, we did not see significant changes in *p* values. Two-tailed *p* values < 0.05 were considered statistically significant.

### Ethics approval

This study was performed in line with the principles of the Declaration of Helsinki. Approval was granted by the local Institutional Review Board of the University Zurich.

## Results

### Clinical characteristics of the study population

We included 55 patients (30 males, 25 females) with a median age (IQR) of 43 (33–53) years. The median BMI was 27 (24–31) kg/m^2^. Three of the 25 female patients were using oral estrogens at the time of the study (one an oral contraceptive pill and two perimenopausal estrogen replacement therapy). In the cohort, five patients had diabetes, one of them had type 1 diabetes. Three were treated with insulin before, and one of them after surgery. The mean (± SEM) creatine levels of the patient from our cohort increased from 66 ± 3 µmol/l to 71 ± 3 µmol/l postoperatively. Out of 55 patients, 39 belonged to group A, 7 to group B, and 9 to group C. Patient characteristics and biomarker levels are summarized in Table 1, which also highlights statistical differences between the groups. The GHR carrier status was most commonly fl/fl (both alleles encoding wild-type GHR) in group A, but not in groups B and C (Table 1).

**Table 1:** Patient characteristics and biomarker levels.

	All (n = 55)	Group A (n = 39)	Group B (n = 7)	Group C (n = 9)	*p* value
Age [years]	43 (33–53)	49 (33–64)	42 (40–45)	37 (29–41)	0.0208
Sex [f%]	45%	44%	43%	56%	> 0.05
BMI [kg/m^2^]	27.3 (24.1–30.8)	27.3 (24.1–29.3)	30.0 (24.5–37.6)	25.7 (24.7–32.4)	> 0.05
Tumor volume [mm^3^]	1642 (440–2948)	1334 (440–2706)	1993 (131–4158)	2278 (917–20,365)	> 0.05
Histological granulation pattern [densely/ sparsely/ diffuse/ n.a.]	28/12/7/8	24/6/4/5	3/2/1/1	1/4/2/2	n.a
MIB-1 staining [< 3%/ > 3%/n.a.]	45/7/3	33/4/2	6/0/1	6/3/0	n.a
GHR (CC/CG/GG/na) [fl/fl;fl/d3;d3/d3;na]	23/24/5/3	20/12/4/3	1/6/0/0	2/6/1/0	n.a
GH pre [µg/l]	15.0 (7.7–38.4)	10.6 (6.6–37.5)	15.0 (5.1–42.6)	24.2 (15.9–64.7)	> 0.05
GH post [µg/l]	1.6 (0.5–3.7)	1.4 (0.4–3.0)*	2.0 (1.4–5.1)*	3.6 (2.2–11.4)*	0.0018
IGF-1 pre [µg/l]	567 (368–707)	560 (356–690)	417 (365–800)	633 (536–723)	> 0.05
IGF-1 post [µg/l]	193 (136–256)	159 (128–227)*	210 (193–256)*	339 (241–490)*	< 0.0001
sKl pre [pg/ml]	3944 (2228–5617)	3123 (1744–5402)	2918 (1986–5617)	5151 (4333–7670)	> 0.05
sKl post [pg/ml]	673 (478–1174)	623 (438–855)*	743 (494–1364)*	1214 (903–5545)*	0.0002

### Pre- and postoperative serum levels of Klotho, GH and IGF-1

**Figure 1:**
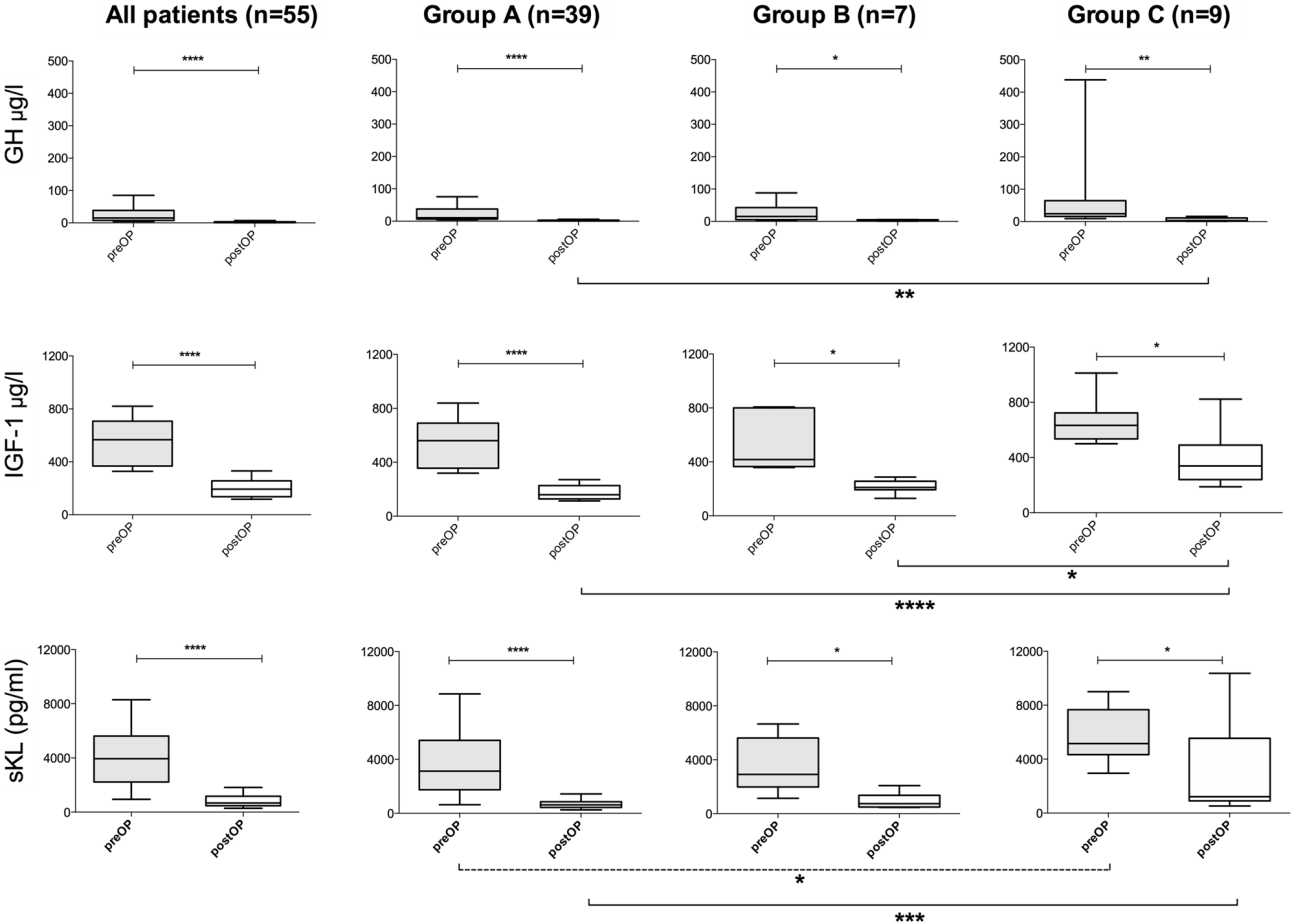
Box plots showing the median (line), the interquartile range (box) and 10th–90th percentiles (whiskers) of preoperative and postoperative (1–3 months after surgery) growth hormone levels, insulin-like growth factor 1 (IGF-1) levels, soluble α-Klotho protein (sKl) levels of all patients (n = 55), for Group A (n = 39), Group B (n = 7), and Group C (n = 9), respectively. Pre- and postoperative GH levels were compared using the Wilcoxon signed rank test. *p < 0.05; **p < 0.01; ***p < 0.001; ****p < 0.0001.

Before surgery, median levels of GH (15, IQR 8–38 µg/l), IGF-1 (567; 368–707 µg/l) and sKl (3944; 2228–5617 pg/ml) were elevated in all groups. Interestingly, preoperative IGF-1 levels were higher in males (634 (482–738) µg/l) than in females (504 (353–617) µg/l; *p* = 0.0384; Mann–Whitney-U test), while preoperative sKl levels were higher in females with 4926 (2672–6820) pg/ml, than in males with 2936 (1531–5348) pg/ml (*p* = 0.0452) (Supplemental Digital Content 1. Figure 1). Pre- and post-operative sKl levels showed a moderate correlation with age (r_s_ = −0.536, *p* < 0.001 and r_s_ = −0.473, *p* < 0.001; Supplemental Digital Content 1. Table 1). Preoperative sKl levels were higher in group C than in groups A and B (Table 1); however, this difference was only statistically significant between groups A and C (*p* = 0.0326, Fig. 1). There was no difference in preoperative concentrations of GH and IGF-1 between the three groups.

After surgery, biomarker values declined statistically significantly to median GH levels of 1.6 (0.5–3.7) µg/l, median IGF-1 levels of 193 (136–256) µg/l, and median sKl levels of 673 (478–1174) pg/ml, respectively (Table 1). Box plots depicting pre- and postoperative values, including statistical testing for all patients and for the three groups separately are shown in Fig. 1. After transsphenoidal pituitary surgery, both median IGF-1 (339; 241–490 µg/l) and sKl (1214; 903–5545 pg/ml) levels remained high in group C. In contrast, there was a substantial decrease in IGF-1, GH and sKI in all patients assigned to group A and group B. Comparing postoperative levels between the subgroups, the difference between postoperative sKl levels between group A and C remained statistically significant (*p* = 0.0007). Additionally, there was a significant difference between GH (*p* = 0.003) and IGF-1 (*p* < 0.0001) concentrations between group A and C. IGF-1 was the only biomarker with a significant difference between groups B and C (*p* = 0.031).

### Relationship between different serum biomarkers with each other and tumor volume

**Figure 2:**
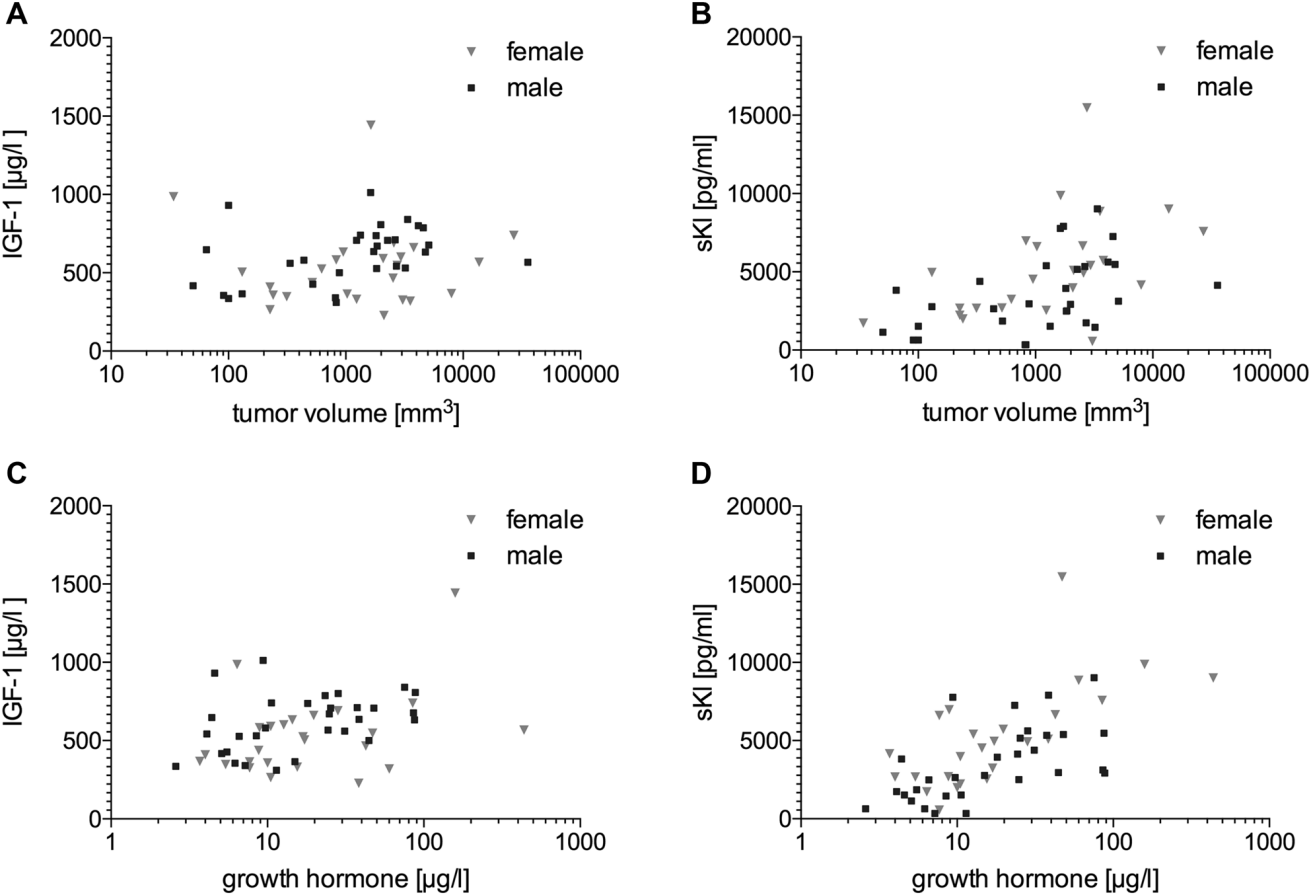
Scatter plots showing the correlation between preoperative values of (**A**) tumor volume (log scale) and insulin-like growth factor 1 (IGF-1, linear scale), r = 0.276, *p* = 0.041; (**B**) tumor volume (log scale) and soluble α-Klotho protein (sKl, linear scale), r = 0.527, *p* < 0.001; (**C**) growth hormone (GH, log scale) and IGF-1 (linear scale), r = 0.368, *p* = 0.006; (**D**) GH (log scale) and sKl (linear scale), r = 0.661, *p* < 0.001; Females are represented by grey triangles and males are shown as black squares. The correlation coefficient r and the p value were calculated according to Spearman.

Preoperative IGF-1 and sKl levels correlated with both basal GH excess and with estimated tumor volume (Fig. 2; Supplemental Digital Content 1. Table 1). However, the correlation with GH and tumor volume was stronger for sKl than for IGF-1. The correlation with GH excess was 0.368 (*p* = 0.006; Spearman`s r) for IGF-1 and 0.661 (*p* < 0.001) for sKl, and with estimated tumor volume 0.276 (*p* = 0.041) for IGF-1 and 0.527 (*p* < 0.001) for sKl. Preoperative sKl levels correlated well with IGF-1 levels before (r = 0.411; *p* = 0.002) and after surgery (r = 0.367; *p* = 0.006; Supplemental Digital Content 1. Figure 2).

### Difference in radiological and histopathological findings between the groups

**Figure 3:**
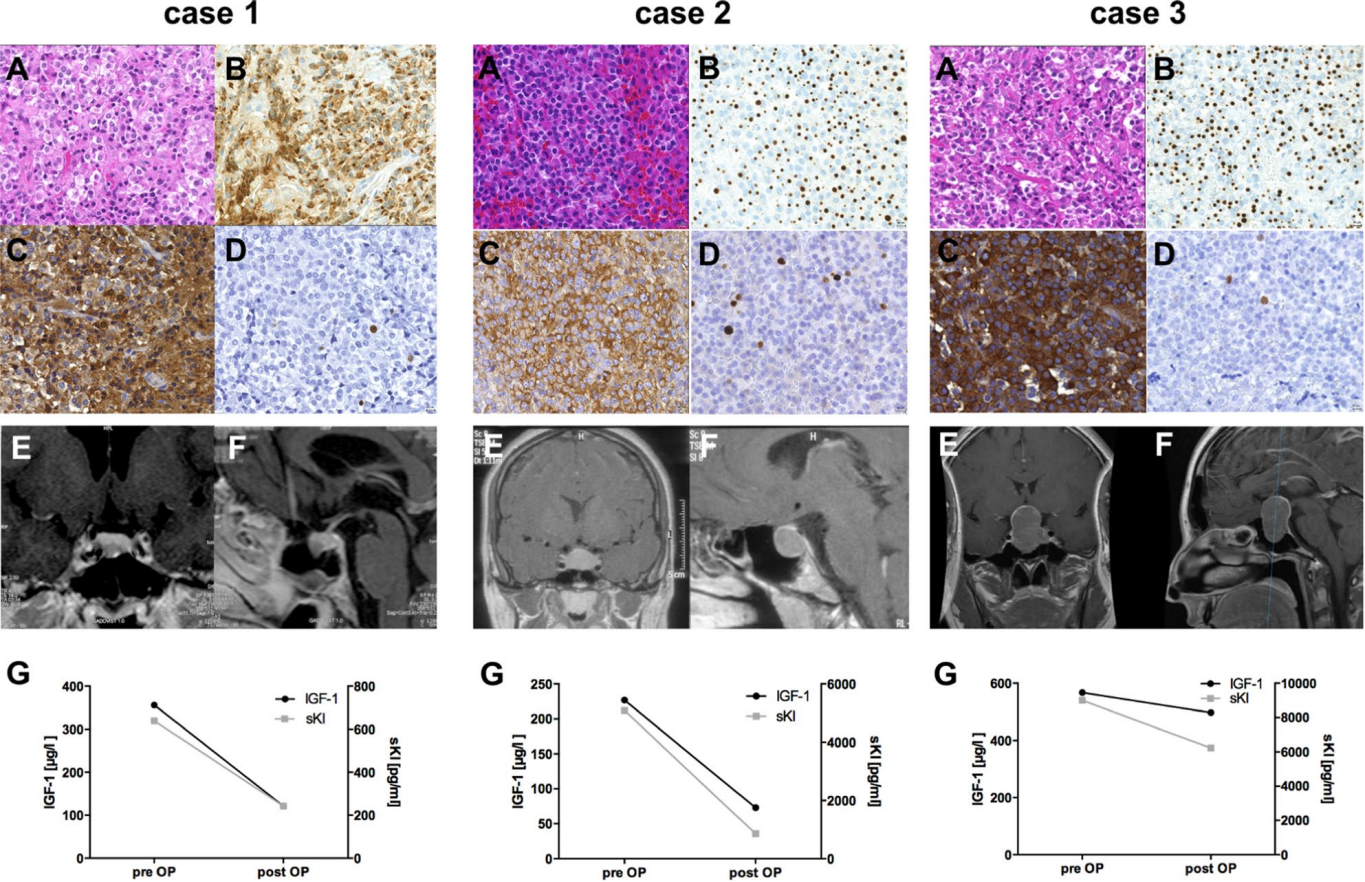
Exemplary cases of patients belonging to different pituitary adenoma subgroups suggested by Cuevas-Ramos et al. Case 1 belongs to Cuevas-Ramos type 1, case 2 to the type 2 group, and case 3 to type 3. Panels (**A**) show H&E staining, (**B**) CK staining (**C**) GH staining, (**D**) MIB-1 staining. Preoperative MR images with gadolinium contrast are depicted in coronal (**E**) and sagittal (**F**) sections. Preoperative and postoperative (1–3 months after surgery) levels of insulin-like growth factor 1 (IGF-1) levels and soluble α-Klotho protein (sKl) are depicted in the bottom row (**G**).

Although preoperative tumor volumes were higher in group C than in group A and B, there was no statistically significant difference between the groups. Regarding suprasellar extension as measured by the Hardy classification, the rate of a Hardy grade above A (suprasellar cistern only) was particularly high in group C (78%) and lower in groups B (43%) and A (24%) (Supplemental Digital Content 1. Figure 3). Similarly, cavernous sinus invasion above a Knosp grade of 1 (no invasion) was more common in group C (56%), than in group B (43%) and group A (18%) (Supplemental Digital Content 1. Figure 4).

The granulation pattern “sparsely granulated” was found in 12/55 (22%) patients. It was least common in group A (15%) patients compared to group B (29%) and C (44%) (Supplemental Digital Content 1. Figure 5). Regarding MIB-1 staining as a marker for cell proliferation, MIB-1 positivity above 3% of cells was rare in our cohort (7/55, 13%) and absent in group B (0/7 patients) (Supplemental Digital Content 1. Figure 6).

We additionally allocated our patients to the novel structural and functional classification system suggested by Cuevas-Ramos and colleagues^26^ (Supplemental Digital Content 1. Figure 6). Strikingly, type 3 adenomas (large, invasive tumors, sparsely granulated) were only represented in groups B (2/7; 29%) and C (3/9; 33%) and never in group A (0/39). Exemplary patients from our cohort belonging to different pituitary adenoma subgroups suggested by Cuevas-Ramos and colleagues are outlined in Fig. 3.

## Discussion

The discovery of sKl excess in acromegaly may serve as an additional marker of disease activity in acromegaly. We have shown in this study that sKl concentrations showed a stronger positive correlation with GH excess and tumor volume than IGF-1 in the pre- and postoperative setting. These results are in line with a published a study by Varewijck et al*.* who showed that IGF-1 bioactivity was related to sKl concentrations and, interestingly, also to quality of life in acromegaly^27^. In addition to serving as a marker reflecting disease activity, sKl might additionally serve as a prognostic marker for remission after transsphenoidal surgery. Considering that approximately 25% of patients do not achieve long-term biochemical remission after surgery, a risk stratification for patients early in the course of their disease may allow for the identification of patients requiring adjuvant medical treatment^15^. Several studies have identified GH and IGF-1 levels as biochemical factors associated with remission after surgery, with GH generally showing a better correlation with postoperative outcome^28–30^. However, due to differences in immunoassays and timing of the biomarker measurements, thresholds associated with remission have not yet been identified. In line with these studies, we have seen a significant difference in postoperative GH and IGF-1 levels between patient in remission and patient with persistent disease activity in our cohort. To our knowledge, this is the first study that additionally showed a significant difference in pre- and postoperative sKl values between patients with long-term biochemical remission and patients with ongoing disease activity. Interestingly, this difference was more pronounced in postoperative values obtained 1–3 months after surgery, suggesting that persistently raised sKl values at early follow-up appointments might identify patients in need of additional therapy.

In light of the well-known technical limitations of the assays for measuring GH and IGF-1, there is an unmet need for more sensitive and specific biomarkers in acromegaly^31^. Serum levels of IGF-1 are known to be influenced by GH status as well as by age, gender, race, liver function, nutritional status, portal insulin, thyroid hormones and inflammatory diseases. Of note, some of these cofounding factors, especially age and gender, may also affect αKl. In addition to these factors, serum IGF-1 is mainly produced the liver and tightly bound to insulin-like growth factor binding proteins (IGFBPs). Changes in IGFBP concentrations contribute to the limitations previously described in some IGF-1 assays^32^. To circumvent these problems, we used a classical and time-consuming assay in which carrier proteins are removed before the samples are incubated with the antibodies^33^. Secondly, regarding the measurement of GH, dynamic testing using oGTT to suppress GH is widely used. However, patients with acromegaly sometimes have normal oGTT GH suppression despite elevated IGF-1 levels^34^. In patients receiving non-surgical treatment of acromegaly such as long-acting somatostatin analogues (LA-SRIFs)^35^, pegvisomant (PEG-V)^36^, or radiotherapy for GH-producing adenomas, GH values may be misleading due to highly irregular GH secretion pattern and flattened GH pulses^37^. In this setting, sKl might serve as an additional useful marker.

In addition to biochemical markers in acromegaly, we compared radiological and histological parameters between patients with long-term remission, recurrence, and persistent disease activity 7–16 years after surgery. In line with previous data, we observed a higher rate of suprasellar extension and cavernous sinus invasion in groups B and C compared to patients in whom long-term biochemical remission was achieved^30,38–40^. In addition, Trouillas and colleagues as well as Cuevas-Ramos and colleagues have suggested novel clinicopathological prognostic and classification scores that integrate invasion and proliferation^26,41,42^. In accordance with these scores, we identified type 3 adenomas according to Cuevas-Ramos (large, invasive tumors, sparsely granulated) exclusively in groups B and C. However, it has to be taken into consideration that these radiological scores are not routinely available in clinical practice. Moreover, there use might be limited due to poor intra- and inter-rater variability, as recently shown for middle scores of the Knosp scale^43^. Thus, their use as predictive markers of remission might be limited compared to biochemical markers.

The data we presented regarding histology and GHR genotype are limited by the small patient cohort. It is feasible that GHR isoforms may affect the interpretation of GH levels, but not outcome. In addition, histological granulation pattern may correlate with growth rate or drug responsiveness in cases with residual tumor. However, GHR-d3 alleles were more frequently observed in group C and group B than in group A (long-term cure) where most patients were homozygous for the wild type receptor alleles. Sparsely granulated adenomas were more common in groups B and C compared to the cure group A. Overall, larger data sets are indicated to investigate the relationship of biochemical outcome with histological features.

Limitations of this study include the retrospective design, which precluded assessment of serum markers at identical time points during follow-up. This especially concerns measurements of IGF-1, which are known to be more reliable 3 months after the surgery. Future prospective studies should therefore aim to schedule follow-up appointments 3–4 months after the surgery, rather than 1–3 months which used to be our routine follow-up period at the time of the study. It is additionally important to highlight that in our cohort, patients with persistent disease activity (group C, n = 9) were younger and tended to have larger tumors than patients with long-term cure (group A, n = 39) even though these differences were not statistically significant across the three groups. A week inverse correlation between age and sKl levels have been described in patients with acromegaly and was also seen in our cohort both before and after surgery. Thus, age could contribute to the particularly high sKl levels observed in group C^44^. Moreover, a complete data set for GH, IGF-1 (assessed by radioimmunoassay after removal of carrier proteins) and αKL (using the same ELISA) obtained by fully identical methods was available only for the perioperative period. Compared to GH and IGF-1 measurements, the assay for αKL was introduced recently, with only limited experience regarding technical shortcomings and physiological ranges.

Although our follow-up periods ranged from 7–16 years, this time frame may still be insufficient to detect late recurrences. While patients belonging to the persistence group are obvious candidates for additional treatment modalities (with subsequent biochemical values subject to alteration by drug treatment), it is important to maintain long-term follow-up for patients with initial remission, who may develop later recurrences.

## Conclusion

Our study showed that sKl may serve as reliable biomarker that reflects disease activity in patients with acromegaly, decreasing markedly after pituitary surgery. In our cohort, it showed superior correlations with GH excess and tumor volume than the well-described biomarker IGF-1. Moreover, sKl may serve as an additional marker of biochemical remission after surgery. To date there are no thresholds that separate the long-term cure group from patients with initial remission and later disease activity. Thus, more studies are needed to define reliable cut-off values in order to optimize the risk stratification of patients with acromegaly.

## Supplementary Information

Supplementary Information.

## Data Availability

The datasets generated during and analysed during the current study are available from the corresponding author on reasonable request.
