# Supplementary material for: Association of pre- and postoperative αKlotho levels with long-term remission after pituitary surgery for acromegaly
Source: Sci Rep. 2022 Aug 30;12:14765. doi: 10.1038/s41598-022-19078-8 (PMC9428163; doi:10.1038/s41598-022-19078-8)
Supplement: Supplementary file 1 — Supplementary Information. [file 41598_2022_19078_MOESM1_ESM.pdf]

Supplemental Table 1 Correlation matrix

	age	BMI	tumor vol.	GH_{pre}	GH_{post}	IGF-1_{pre}	IGF-1_{post}	sKl_{pre}	sKl_{post}
Age	-	0.129 (0.348)	**-0.291 (0.031)**	**-0.368 (0.006)**	0.036 (0.793)	-0.200 (0.144)	**-0.424 (0.001)**	**-0.536 (<0.001)**	**-0.473 (<0.001)**
BMI	0.129 (0.348)	-	-0.078 (0.571)	-0.035 (0.799)	-0.165 (0.229)	**0.414 (0.002)**	0.188 (0.168)	-0.068 (0.623)	-0.100 (0.469)
tumor vol.	**-0.291 (0.031)**	-0.078 (0.571)	-	**0.557 (<0.001)**	**0.309 (0.022)**	**0.276 (0.041)**	0.160 (0.243)	**0.527 (<0.001)**	0.257 (0.058)
GH_{pre}	**-0.368 (0.006)**	-0.035 (0.799)	**0.557 (<0.001)**	-	**0.281 (0.038)**	**0.368 (0.006)**	**0.314 (0.020)**	**0.661 (<0.001)**	**0.416 (0.002)**
GH_{post}	0.036 (0.793)	-0.165 (0.229)	**0.309 (0.022)**	**0.281 (0.038)**	-	-0.003 (0.980)	**0.322 (0.016)**	0.087 (0.526)	**0.269 (0.047)**
IGF-1_{pre}	-0.200 (0.144)	**0.414 (0.002)**	**0.276 (0.041)**	**0.368 (0.006)**	-0.003 (0.980)	-	**0.510 (<0.001)**	**0.411 (0.002)**	0.133 (0.334)
IGF-1_{post}	**-0.424 (0.001)**	0.188 (0.168)	0.160 (0.243)	**0.314 (0.020)**	**0.322 (0.016)**	**0.510 (<0.001)**	-	0.254 (0.061)	**0.367 (0.006)**
sKl_{pre}	**-0.536 (<0.001)**	-0.068 (0.623)	**0.527 (<0.001)**	**0.661 (<0.001)**	0.087 (0.526)	**0.411 (0.002)**	0.254 (0.061)	-	**0.695 (<0.001)**
sKl_{post}	**-0.473 (<0.001)**	-0.100 (0.469)	0.257 (0.058)	**0.416 (0.002)**	**0.269 (0.047)**	0.133 (0.334)	**0.367 (0.006)**	**0.695 (<0.001)**	-

Correlation matrix showing Spearman`s correlation coefficient and the p-value in brackets (two-sided). Statistically significant p-values are depicted in **bold**.

abbreviations: BMI = body mass index; GH= growth hormone; IGF-1 = insulin-like growth factor 1; sKl = soluble alpha-Klotho; pre = preoperative values; post = early postoperative values (1-3 months following surgery)

Supplemental Figure 1

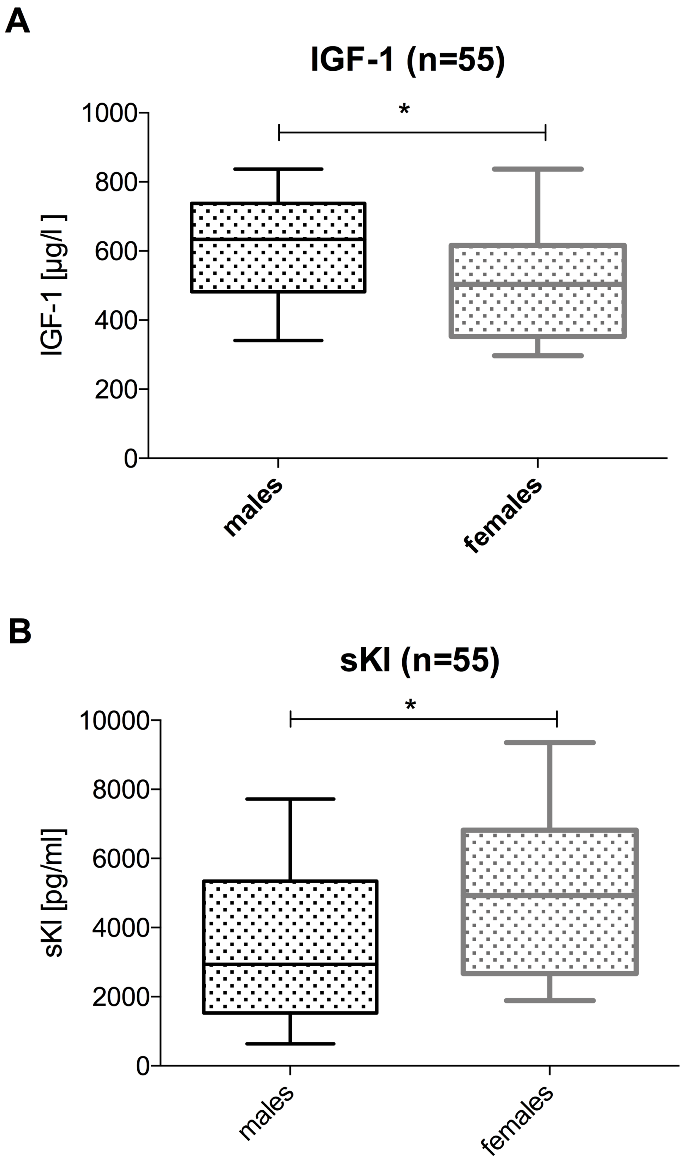

Box plots showing the median (line), the interquartile range (box) and 10th – 90th percentiles (whiskers) of preoperative insulin-like growth factor 1 (IGF-1) levels and soluble α-Klotho protein (sKl) levels in male and female patients. * $p < 0.05$; ** $p < 0.01$; *** $p < 0.001$; **** $p < 0.0001$

Supplemental Figure 2

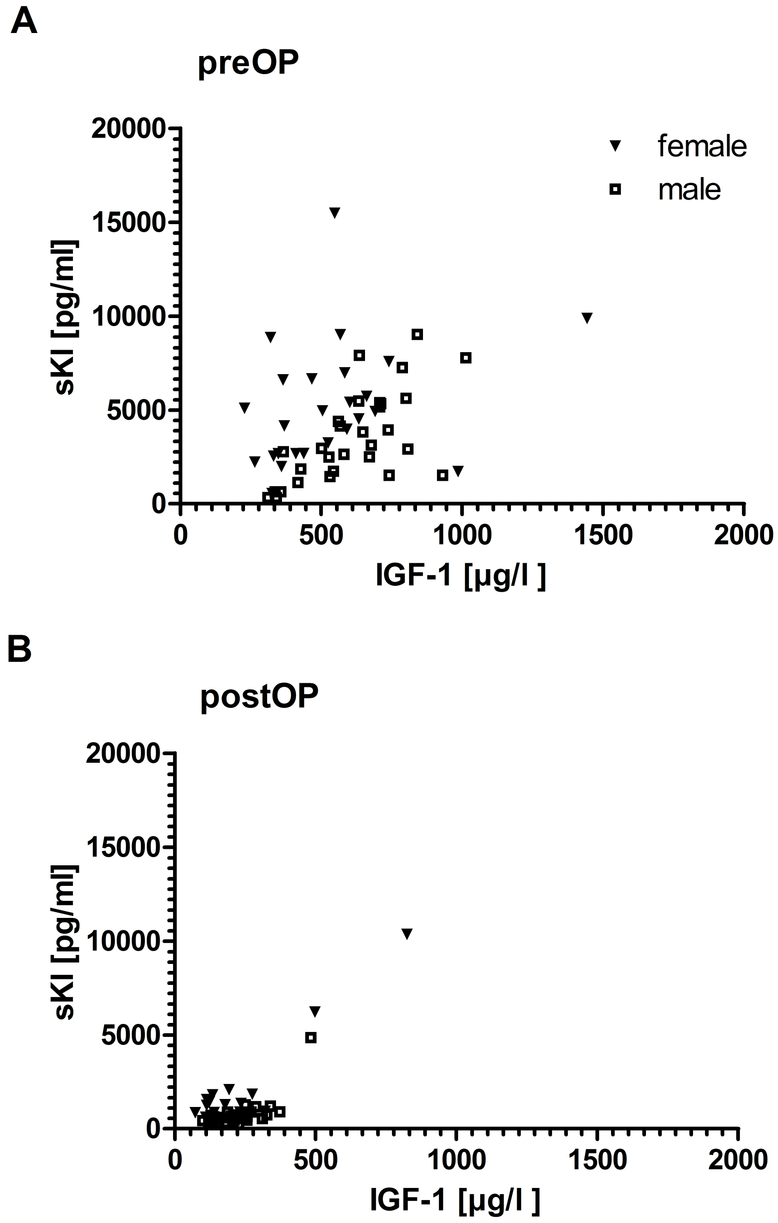

Scatter plots showing the correlation between (**A**) preoperative values of IGF-1 (linear scale) and sKl (linear scale), r=0.411, p=0.002 as well as (**B**) postoperative values (1-3 months following surgery) of the two markers, r=0.367, p=0.006. Females are

represented by black triangles and males are shown as white squares. The correlation coefficients r and the p values were calculated according to Spearman.

Supplemental Figure 3

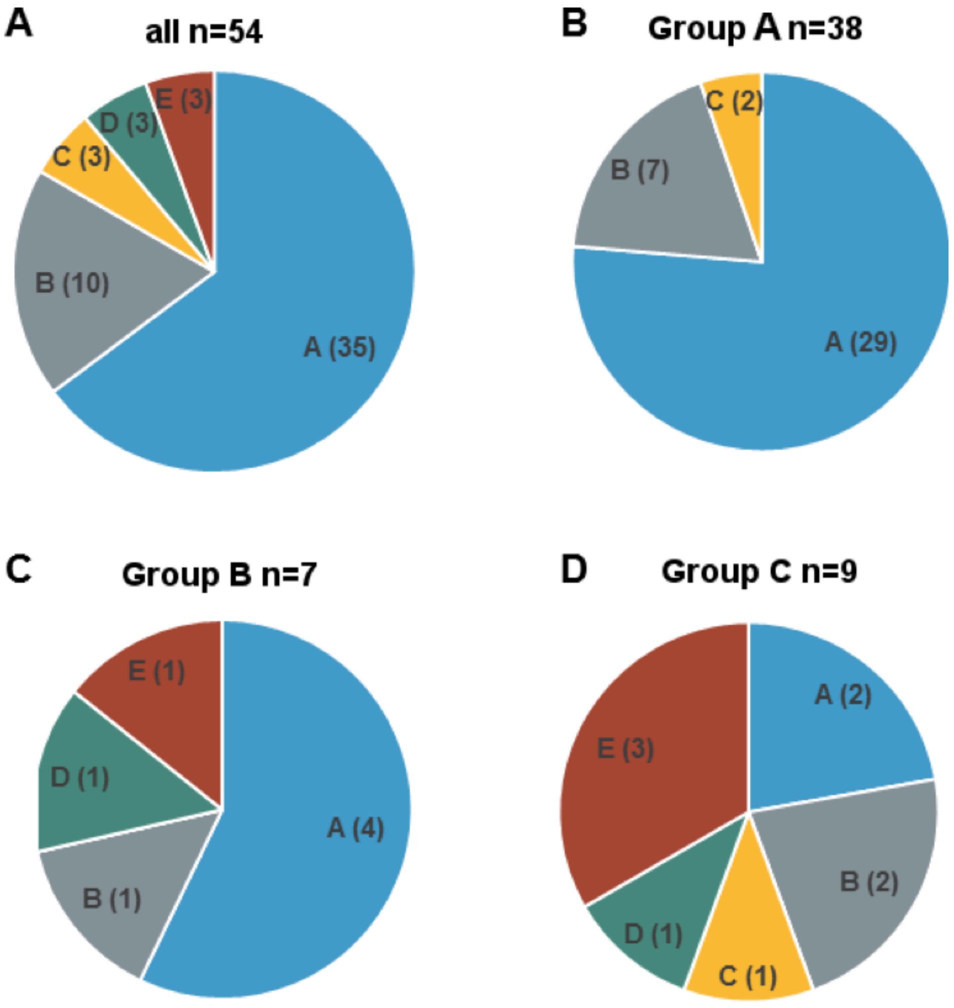

Hardy classification system for all patients (**A**), group A (**B**), group B (**C**), and group C (**D**). Symmetrical adenoma (grade A: suprasellar cistern only; grade B: recess of the third ventricle; or grade C: whole anterior third ventricle) or asymmetrical adenoma (grade D, intracranial extradural; or grade E, extracranial extradural [cavernous sinus]). One patient (belonging to group A) could not be classified based on the available preoperative imaging data.

Supplemental Figure 4

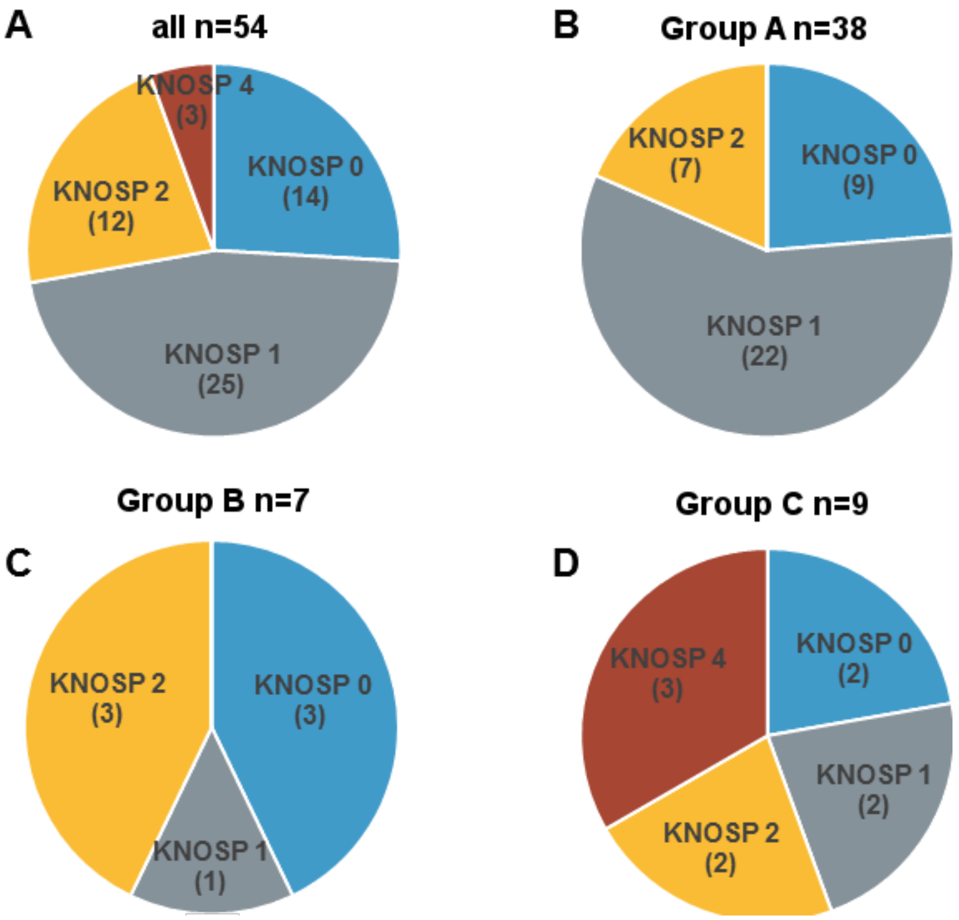

Knosp classification system to quantify cavernous sinus invasion for all patients (**A**), group A (**B**), group B (**C**), and group C (**D**). Grade 0: no cavernous sinus involvement; grade 1 and 2: the tumor pushes into the medial wall of the cavernous sinus, but does not go beyond a hypothetical line extending between the centers of the two segments of the internal carotid artery (grade 1) or it goes beyond such a line, but without passing a line tangent to the lateral margins of the artery itself (grade 2); grade 3: the tumor extends laterally to the internal carotid artery within the cavernous sinus; grade 4: total encasement of the intracavernous carotid artery. One patient (belonging to the group A) could not be classified based on the available preoperative imaging data.

Supplemental Figure 5

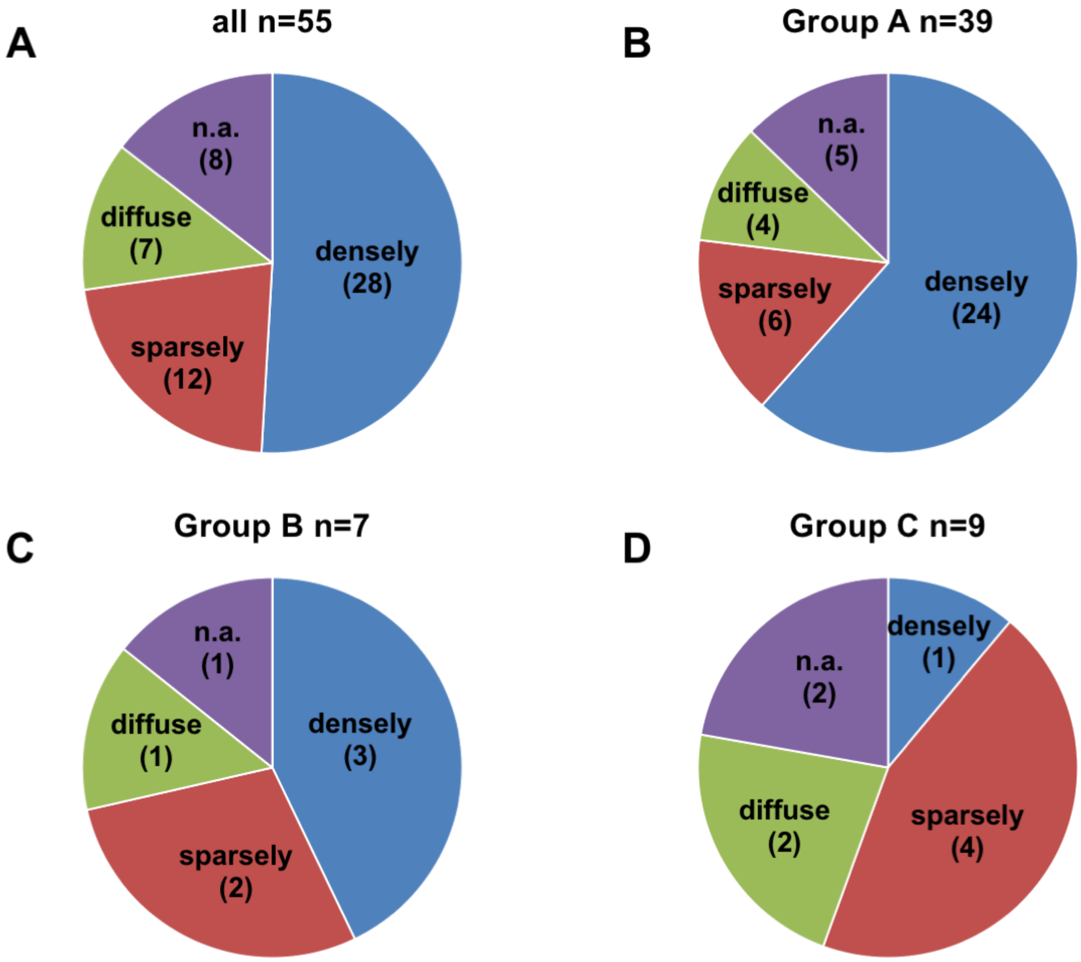

Histological granulation pattern for all patients (**A**), group A (**B**), group B (**C**), and group C (**D**).

Supplemental Figure 6

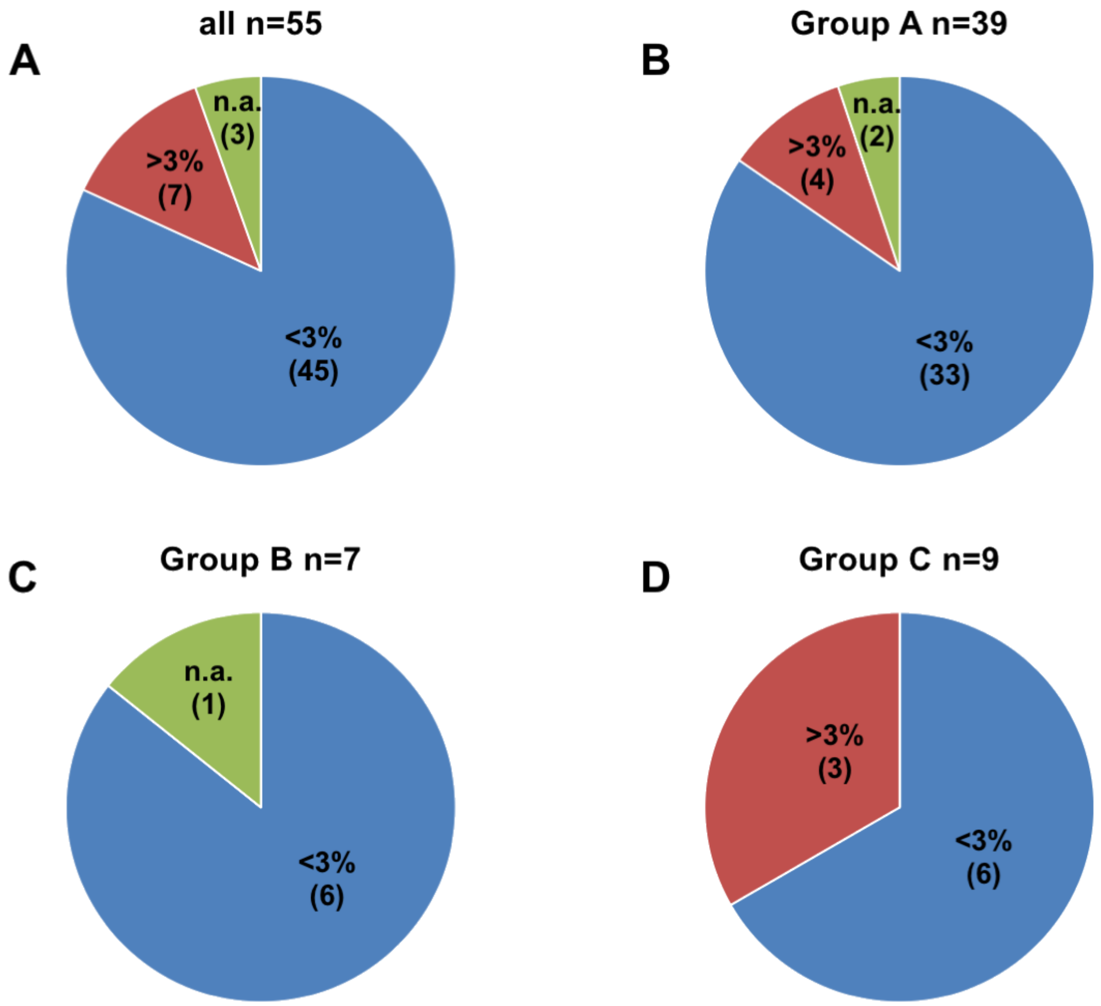

MIB1 immunostaining for all patients (**A**), group A (**B**), group B (**C**), and group C (**D**).

Supplemental Figure 7

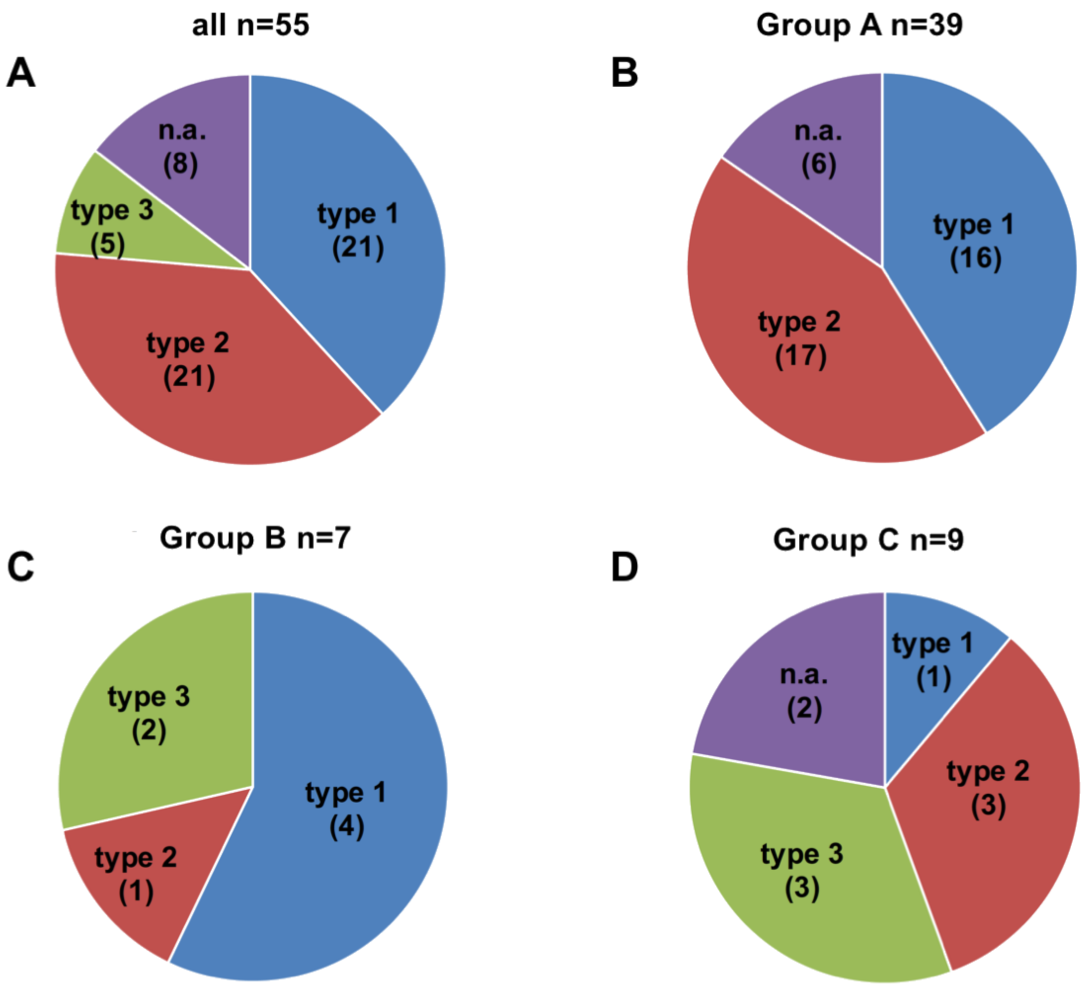

Patient allocation to Cuevas-Ramos groups for all patients (**A**), group A (**B**), group B (**C**), and group C (**D**).
